# Forearm T-score as a predictor of cage subsidence in patients with degenerative lumbar spine disease following posterior single-segment lumbar interbody fusion

**DOI:** 10.1186/s12891-022-05930-5

**Published:** 2022-12-05

**Authors:** Hong-yu Pu, Qian Chen, Kun Huang, Rui Zeng, Peng Wei

**Affiliations:** 1 Department of Orthopaedic Surgery, the Affiliated Hospital of North Sichuan Medical College, Nanchong, 637000 China Sichuan Province; 2 The Affiliated Traditional Chinese Medicine Hospital of Southwest Medical University, Luzhou, 646000 Sichuan Province China

**Keywords:** Osteoporosis, Cage subsidence, Forearm T-score, Hounsfield units, Dual-energy absorptiometry

## Abstract

**Background:**

Posterior lumbar interbody fusion (PLIF) has become a classic treatment modality for lumbar degenerative diseases, with cage subsidence as a potentially fatal complication due to low bone mineral density (BMD), which can be measured by forearm T-score. Hounsfield units (HU) derived from computed tomography have been a reliable method for assessing BMD.

**Objective:**

To determine the accuracy of forearm T-score in predicting cage subsidence after PLIF compared with lumbar spine HU values.

**Methods:**

We retrospectively analyzed the clinical data of 71 patients who underwent PLIF and divided them into cage subsidence group and nonsubsidence group. The differences in preoperative HU value and forearm T-score were compared between groups, and the correlation between cage subsidence and clinical efficacy was analyzed.

**Results:**

The subsidence rate for all 71 patients (31 men and 40 women) was 23.9%. There was no significant difference in age, sex ratio, body mass index, smoking status, follow-up time, spine BMD, and spine T-score between groups, except in the forearm T-score and lumbar spine HU values (*P* < 0.05). The forearm T-score (AUC, 0.840; 95% CI, 0.672–1.000) predicted cage subsidence more accurately than the mean global HU value (AUC, 0.744; 95% CI, 0.544–0.943). In logistic regression analysis, both forearm T-score and mean global HU value were found to be independent risk factors for cage subsidence (*P* < 0.05).

**Conclusions:**

Lower forearm T-scores and lower lumbar spine HU values were significantly associated with the occurrence of cage subsidence. Lower forearm T-scores indicated a higher risk of cage subsidence than lumbar spine HU values. Forearm T-score is more effective in predicting cage subsidence than spine T-score. Therefore, forearm dual-energy X-ray absorptiometry may be a fast, simple, and reliable method for predicting cage subsidence following PLIF. However, our results suggest that the degree of cage subsidence is not associated with clinical efficacy.

## Background

Posterior lumbar interbody fusion (PLIF) is a classic surgical approach for the treatment of degenerative lumbar spine diseases, as it provides load burden on the anterior column of the spine, adequate decompression of nerve roots, and restoration of intervertebral space height [1, 2]. However, its common long-term complications include cage subsidence, adjacent spondylosis, and pseudoarthrosis [3]. Cage subsidence can lead to lordosis and decrease foraminal height, thereby aggravating postoperative low back pain and recurrent lower extremity neurological symptoms. As low bone density is an important risk factor for cage subsidence [4, 5], preoperative measurement of bone mineral density (BMD) is crucial for predicting this complication.

Osteoporosis is mainly diagnosed using dual-energy X-ray absorptiometry (DXA) by measuring the lumbar spine BMD. However, BMD is usually overestimated via lumbar DXA in patients with lumbar degenerative disease. The International Society for Clinical Densitometry (2019) recommends measuring BMD in the distal forearm when measurement or interpretation of the hip and/or spine is not possible. BMD of the forearm has higher accuracy and sensitivity in screening for osteoporosis than BMD of other sites [6–8]. In contrast, the diagnostic accuracy of vertebral body Hounsfield units (HU) values for osteoporosis has recently gained widespread acceptance and has become a new method for measuring BMD. In addition, vertebral body HU values are correlated with T-scores [4, 9, 10]. However, the method of measuring the HU value is less popular and thus not standardized. Therefore, the BMD of the distal forearm is still measured clinically to screen patients for osteoporosis. As the relationship between forearm BMD and cage subsidence has not been reported, this study aimed to determine the relationship between forearm BMD and cage subsidence following PLIF combined with bilateral pedicle screw internal fixation.

## Methods

### Patient population

This study was approved by our Institutional Review Board. Clinical data of 71 patients admitted to an orthopedic department for PLIF combined with bilateral pedicle screw fixation between January 1, 2020, and June 30, 2021, were retrospectively analyzed. The inclusion criteria were as follows: (1) lumbar spinal stenosis (including foraminal stenosis) and degenerative spondylolisthesis; (2) PLIF combined with bilateral pedicle screw fixation only at the level of L4–L5; and (3) complete imaging and clinical data, including lumbar spine X-rays before surgery, 1 week after surgery, and last follow-up (at least 12 months) and lumbar spine CT, forearm DXA measurements, and lumbar spine DXA measurements at the last follow-up (at least 12 months). The exclusion criteria were as follows: (1) any history of spinal surgery; (2) history of spinal diseases such as spinal tuberculosis, spinal tumor, and ankylosing spondylitis; (3) DXA of the dominant hand forearm; (4) the presence of nondominant forearm and wrist fracture; and (5) history of surgery.

### Dual-energy X-ray absorptiometry

DXA was preoperatively performed to measure BMD (measured in g/cm^2^) of each patient at the distal 1/3 of the length of the ulna and radius of the nondominant forearm and the L1 vertebral body. Fixation was performed by a physician certified in International Clinical Bone Measurements. BMD was examined using the EXA-3000 Osteosys absorptiometry system (X-ray absorptiometry), which was preheated and calibrated before testing. The detection parameters included scanning current at 0.25 mA and tube voltage at 80 kV/55 kV, and the parameters were set during the detection. According to the World Health Organization standard classification, osteoporosis is defined as T-score ≤ − 2.5; osteopenia as − 2.5 < T-score < − 1; and normal BMD as T-score ≥ − 1.0.

### Radiographic measurements

**Fig. 1:**
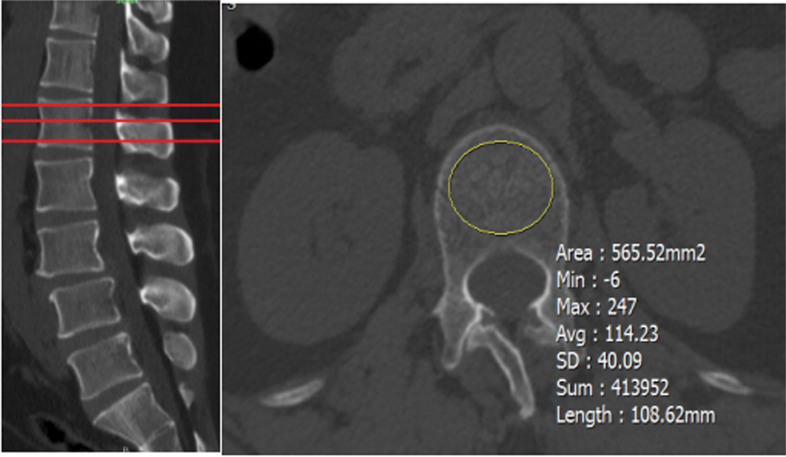
The left panel indicates the selection of the middle L1 vertebral body in the sagittal position of the CT image, and the right panel indicates the placement of the ROI in the mid-level axial position of the L1 vertebral body and the automatic calculation of the mean HU value within the ROI by the PACS system

All computed tomography (CT) scans were performed using dual-source CT (SOMATOM Definition). As the CT window type does not change the HU value, preoperative CT was routinely used to calculate HU values. HU values were measured by placing an oval region of interest (ROI) on three images of the L1 to L5 vertebral body axially, slightly below the superior endplate, in the middle of the vertebral body, and slightly above the inferior endplate. For each measurement, the largest possible elliptical area of interest was drawn, and the cortical edge was excluded. The standard picture archiving and communication system (PACS) automatically calculates the average HU value for the ROI. The average HU values of the three regions of interest were obtained as the BMD of the corresponding vertebral trabecular bone (Fig. 1). The mean global HU value was defined as the average HU value of L1 to L5, and the operated segment HU value was the average HU value of L4 and L5. To place the ROI, we included as much trabecular bone as possible and avoided cortical bone and areas of heterogeneity such as the posterior venous plexus, bony islands, and compressed bone.

Anterior–posterior and lateral X-rays of the lumbar spine were obtained at 1 week postoperatively and at the last follow-up (at least 12 months); three-dimensional CT was also performed at the last follow-up. The distance of cage subsidence on CT mid-sagittal images were measured. Considering that the cage may enter the endplate nonparallel to the intervertebral space, cage subsidence was defined as the maximum distance of displacement of the cage into the cranial or caudal endplate by > 2 mm. Depending on whether the cage subsided by ≥2 mm, the patients were divided into the subsidence (≥2 mm) and nonsubsidence (< 2 mm) groups. All radiological parameters were measured by two independent observers (a researcher and an orthopedic surgeon) who were blinded to the patients’ DXA measurements.

### Clinical measurements

The visual analog scale (VAS) scores of back and leg pain and lumbar spine Japanese Orthopaedic Association (JOA) scores were recorded before surgery, 1 week after surgery, and at the last follow-up. The improvement rate of lumbar spine JOA score = (postoperative lumbar spine JOA score − preoperative lumbar spine JOA score) / (total score − preoperative lumbar spine JOA score) × 100%.

### Statistical analysis

Statistical analysis was performed using SPSS version 26 (SPSS, USA). Continuous variables were analyzed using independent samples t-test, whereas categorical variables were analyzed using the chi-square test or Fisher’s exact test. Binary logistic regression analysis was performed to determine the influencing factors of cage subsidence, and the results were expressed as odds ratios (ORs) with 95% confidence intervals. Intraclass correlation coefficients (ICCs) were used to assess the inter- and intra-observer reliability of HU and cage subsidence measurements (an ICC of ≥0.8 indicated excellent reliability). The receiver operating characteristic (ROC) curve and area under the curve (AUC) were used to determine the thresholds of factors influencing cage subsidence. Pearson correlation coefficient was used to analyze the correlation between the degree of cage subsidence and VAS score and improvement rate of lumbar JOA score. A two-sided significance level of α = 0.05 was considered.

## Results

This study included 71 patients (31 men and 40 women; mean age, 59.6 ± 10.1 years) who underwent PLIF treatment. The mean body mass index (BMI) was 25.8 ± 4.1 kg/m^2^, the mean follow-up time was 13.6 ± 5.1 months, and the subsidence rate was 23.9% (*n* = 17). The main diagnosis was lumbar spinal stenosis and degenerative spondylolisthesis. Between the subsidence and nonsubsidence groups, there was no significant difference in sex ratio, BMI, smoking status, follow-up time, spine BMD, and spine T-score (*P* > 0.05). Demographic characteristics and bone density measured using DXA or HU value are summarized in Table 1.

**Table 1:** Demographic characteristics and bone density

	Subsidence group (*n* = 17)	Non-subsidence group (*n* = 54)	*P* value
Age (y)	59.2 ± 9.8	59.9 ± 10.9	0.873
Gender ratio (male:female)	7:10	24:30	0.927
Smoking (yes/no)	3:14	9:45	0.965
Follow-up time (m)	12.3 ± 5.8	12.7 ± 4.6	0.591
BMI (kg/m^2^):	26.0 ± 5.4	25.6 ± 2.7	0.842
Spine BMD (g/cm^2^)	0.845 ± 0.147	0.885 ± 0.168	0.535
Spinal T-score:	−1.8 ± 1.3	−1.6 + 1.1	0.476
Forearm BMD (g/cm^2^)	0.284 ± 0.662	0.429 ± 0.956	<0.001
Forearm T-score	−2.7 ± 1.1	−1.2 ± 1.2	<0.001
Mean global HU value	96.1 ± 45.0	132.7 ± 40.2	0.015
Operated segment HU value	96.7 ± 55.2	129.0 ± 42.2	0.038
Upper vertebrae of operated segment HU value	94.4 ± 44.6	129.6 ± 40.7	0.029
Lower vertebrae of operated segment HU value	98.5 ± 55.9	130.1 ± 44.4	0.033

### HU value and cage subsidence

**Fig. 2:**
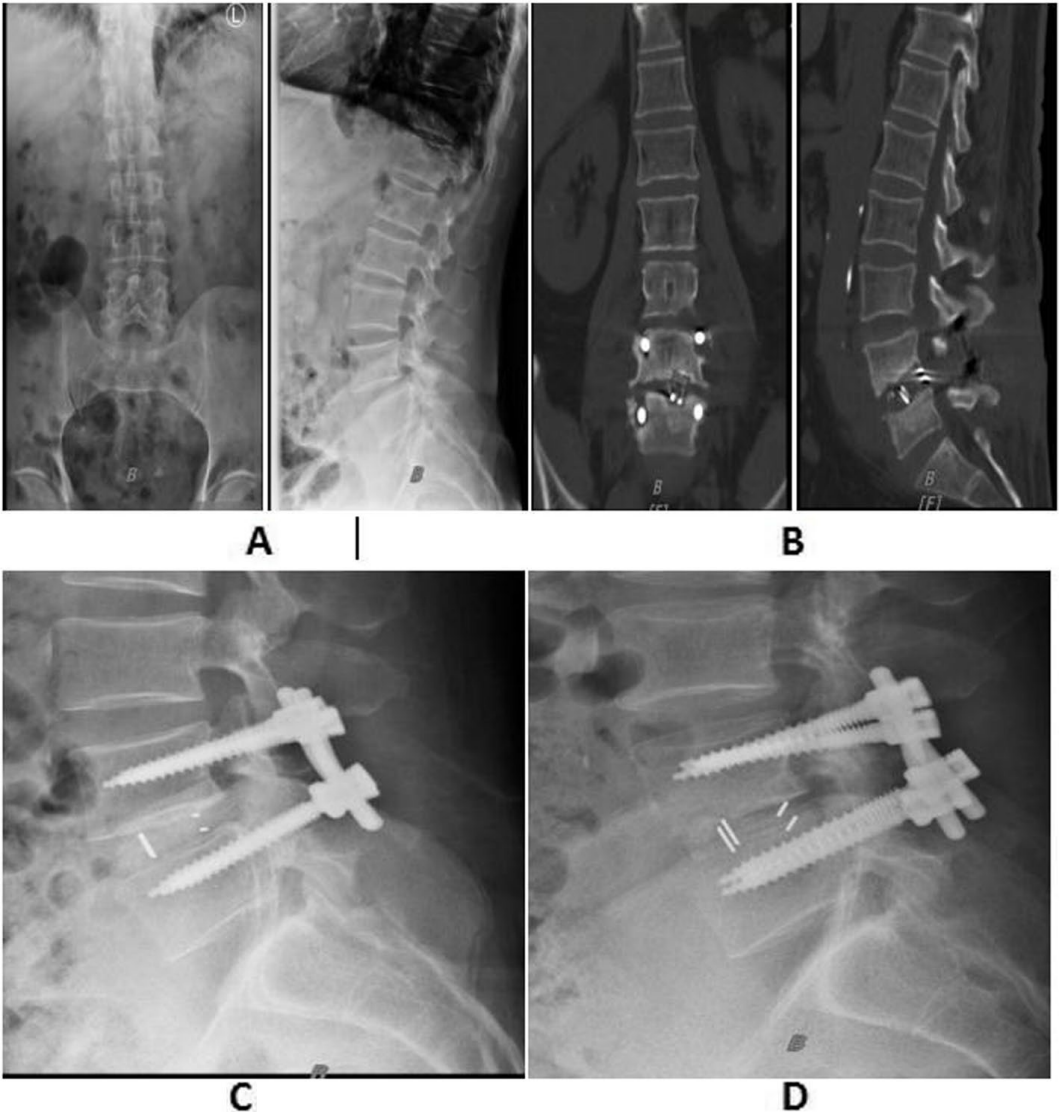
A 66-year-old osteoporotic woman with lumbar spondylolisthesis (forearm T-score = − 2.8) underwent PLIF. **A** Preoperative anterior–posterior and lateral lumbar X-rays. **B** Measurement of cage subsidence distance on CT mid-sagittal and coronal images at 15 months postoperatively. **C** Lateral lumbar X-rays at 1 week postoperatively. **D** Cage subsidence on X-ray at 1 year postoperatively

**Fig. 3:**
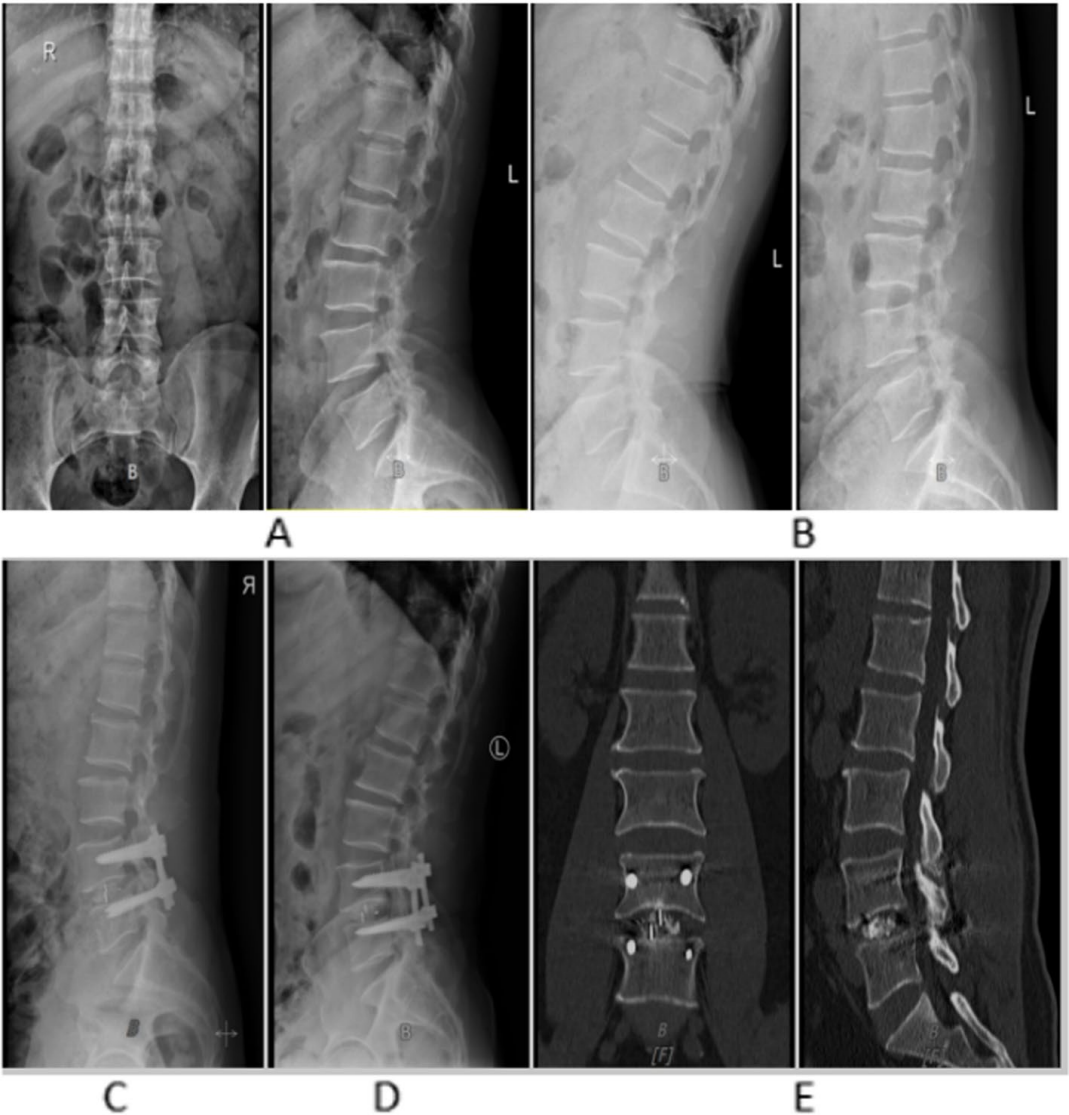
A 56-year-old nonosteoporotic man with lumbar spinal stenosis (forearm T-score = − 1.5) underwent PLIF. **A** Preoperative anterior–posterior and lateral lumbar X-rays. **B** Preoperative flexion and extension lumbar X-rays. **C** Lateral lumbar X-ray at 1 week postoperatively. **D** and **E** No subsidence of the cage was observed in X-ray and CT images at 1 year postoperatively

The intra- and inter-observer reliability of measuring HU values and cage subsidence was very good, with ICC of 0.912 and 0.933 for measuring HU values, respectively, and 0.945 and 0.953 for measuring cage subsidence, respectively. The HU values (mean global HU value, operated segment HU value, upper vertebrae of operated segment HU value, and lower vertebrae of operated segment HU value) were significantly different between the two groups (*P* < 0.05). In the ROC curve analysis (Table 2), among these HU values, the mean global HU value had the largest AUC and could significantly predict cage subsidence (AUC, 0.744; 95% CI, 0.544–0.943). Figures 2 and 3 show representative examples in the subsidence and nonsubsidence groups, respectively, such as preoperative X-rays in static (anterior–posterior and lateral) and dynamic (flexion and extension) modes, lateral X-ray 1 week after surgery, and lateral X-rays and CT at the last follow-up.

**Table 2:** Results of ROC analysis

	AUC (95%CI)	*P* value	Cut off (HU)	Sensitivity (%)	Specificity (%)
Upper vertebrae of operated segment HU value:	0.724(0.521–0.928)	0.042	98.7	0.769	0.667
Lower vertebrae of operated segment HU value:	0.702(0.483–0.921)	0.032	96.6	0.923	0.583
Mean global HU value:	0.744(0.544–0.943)	0.004	104.2	0.846	0.664
Operated segment HU value:	0.718(0.504–0.932)	0.027	97.2	0.925	0.580
Forearm T-score:	0.840(0.672–1.000)	0.004	−2.6	0.846	0.883

### T-score and cage subsidence

**Fig. 4:**
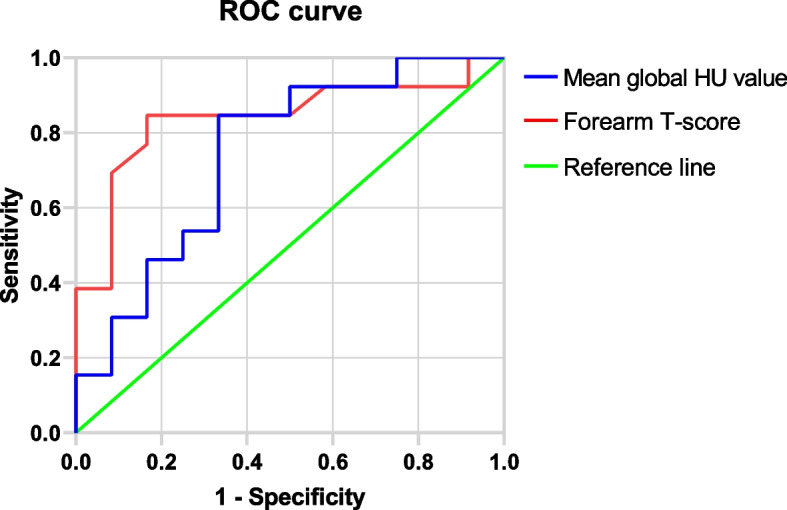
An ROC curve for evaluating the mean global HU values, forearm T-scores, and cage subsidence

The spinal T-score was not significantly different between the two groups (− 1.8 ± 1.3 vs. − 1.6 ± 1.1, *P* > 0.05). In contrast, the forearm T-scores were significantly different (− 2.7 ± 1.1 vs. − 1.2 ± 1.2, *P* < 0.001). In the ROC curve analysis (Table 2), compared with the mean global HU value (AUC, 0.744; 95% CI, 0.544–0.943), the forearm T-score (AUC, 0.840; 95% CI, 0.672–1.000) was more accurate in predicting cage subsidence (Fig. 4). A forearm T-score of − 2.6, with balanced sensitivity of 84.6% and specificity of 88.3%, was set as the cutoff value for identifying patients at higher risk of cage subsidence. The cutoff value of the mean global HU value was 104.2, with a balanced sensitivity of 84.6% and specificity of 66.4% (*P* < 0.001).

### T-score and HU value

The related factors with *P* < 0.1 compared between the two groups in Table 1 were included in the logistic regression analysis as possible factors influencing cage subsidence, including the forearm T-score and HU value of the lumbar spine. Based on the AUC of the operated segment HU value, the upper and lower vertebrae of operated segment HU value were highly consistent with the mean global HU value, which had the largest AUC value and thus selected for analysis. In the logistic regression analysis, the forearm T-score and mean global HU value were found to be independent risk factors for cage subsidence (*P* < 0.05) (Table 3).

**Table 3:** Logistic regression analysis of the factors affecting cage subsidence

Variable	β	Standard error	*P* value	OR
Mean global HU value	−0.132	0.102	0.031	0.752
Forearm T-score	−1.118	1.657	0.016	0.884

### Clinical outcomes

Between the subsidence and nonsubsidence groups, no significant difference was noted in the VAS score and improvement rate of JOA score before surgery, 1 week after surgery, and at the last follow-up (*P* > 0.05) (Table 4). The Pearson test showed that the degree of cage subsidence at the last follow-up showed no significant correlation with the VAS score (back) (*P* = 0.233), VAS score (leg) (*P* = 0.462) or the improvement rate of JOA score (*P* = 0.116). Thus, the VAS score and the improvement rate of JOA score were not significantly correlated with the degree of cage subsidence.

**Table 4:** Comparison of clinical results between the subsidence and nonsubsidence groups

	Clinical outcomes	Subsidence group	Non-subsidence group	*P* value
Preoperatively	VAS (back)	6.1 ± 0.5	6.2 ± 0.4	0.621
	VAS (leg)	6.9 ± 0.8	7.0 ± 0.8	0.235
Postoperative at 1 week	VAS (back)	2.5 ± 0.7	2.4 ± 0.8	0.459
	VAS (leg)	3.2 ± 0.6	3.4 ± 0.9	0.177
	the improvement rate of JOA score (%)	55.45 ± 17.53	57.43 ± 20.66	0.651
Last follow-up	VAS (back)	1.2 ± 0.2	1.3 ± 0.3	0.472
	VAS (leg)	1.6 ± 0.6	1.8 ± 0.5	0.191
	the improvement rate of JOA score (%)	73.26 ± 23.34	76.42 ± 25.78	0.534

## Discussion

PLIF was first proposed by Cloward in the 1940s [9], and it has now become the standard treatment for lumbar degenerative diseases. However, cage subsidence is a common complication after PLIF, induced by low bone density, endplate damage, too small and pre-placed cage, and excessive distraction of the intervertebral space [10]. Except for low BMD, which can be measured preoperatively, other factors are closely related to surgical technique and intraoperative selection. Therefore, BMD testing should be routinely performed before lumbar fusion surgery, and active antiosteoporosis treatment should be performed for patients with osteoporosis to reduce the occurrence of postoperative cage subsidence.

At present, the definition of cage subsidence has not been fully standardized. Marchi et al. [4] graded cage subsidence according to the ratio of the reduction in the height of the intervertebral space on lateral lumbar radiographs. However, after lumbar fusion, a certain reduction in the height of the intervertebral space is considered a normal process of endplate remodeling due to aggressive discectomy [11]. Therefore, the grade 0 description in this classification method does not clearly distinguish between subsidence immediately after surgery and cage subsidence due to low bone density. Considering the possible subsidence of the cage that is not parallel to the intervertebral space, cage subsidence has been defined as the displacement of the cage toward the rostral or caudal endplate by > 2 mm on CT sagittal images [4, 12, 13]. It may be more accurate to measure the difference in distance between cages entering the endplate on postoperative and late CT scans, but postoperative CT was not routinely obtained in the present study. Therefore, the measurements derived by CT sagittal images in this study were consistent with those of previously published methods [4, 12, 14].

The correlation between cage subsidence and postoperative clinical efficacy has been controversial. Cho et al. [15] conducted a 2-year follow-up of 55 osteoporotic and nonosteoporotic patients who received PLIF and found that, compared with the nonosteoporotic group, the osteoporosis group had a higher incidence of cage subsidence, but without significant difference in the improvement of clinical symptoms. Similarly, Oh [5] and Satake [16] also found no significant difference in postoperative clinical efficacy between the cage subsidence and nonsubsidence groups. However, Tohmeh et al. [17] believed that cage subsidence would significantly affect clinical efficacy after surgery. When the subsidence was > 4 mm, the postoperative Oswestry Disability Index, quality of life assessment, and VAS scores for low back pain were significantly worse. Similarly, Marchi et al. [18] suggested that early postoperative cage subsidence would induce transient low back pain. Despite the high incidence of cage subsidence rate at 23.9% in the present study, clinical efficacy between groups was not significantly different. Because cage subsidence usually occurs within 3 months after surgery, it is recommended that patients wear a lumbar brace for more than 3 months.

HU value can be used to selectively measure the bone density of cancellous bone to avoid the degeneration area, thereby improving the diagnostic accuracy. Zhou et al. [19] compared the accuracy of T-score and lumbar HU values in predicting cage subsidence and found that the preoperative HU value of the lumbar spine was more accurate, but the T-score in the present study was obtained by measuring the lumbar spine using DXA. However, because of lumbar spondylolisthesis, intervertebral space stenosis, osteophyte formation, osteosclerosis, and abdominal wall vascular calcification, BMD was overestimated by the T-score obtained using lumbar DXA [20]. Rey et al. [21] and Bina et al. [7] compared the forearm DXA measurements with lumbar spine DXA measurements and reported a significant linear correlation between forearm BMD and lumbar spine BMD and that forearm DXA was useful in diagnosing osteoporosis in postmenopausal women, exhibiting better accuracy than lumbar DXA. In addition, Pouillès et al. [8] found that DXA measurement of the forearm is an effective tool for OP screening and can directly identify approximately 50% of patients without central OP. Moreover, forearm DXA is a fast, inexpensive, and low-radiation skeletal state assessment method [22], which is widely used in clinical practice. In this study, BMD of the distal forearm was measured using DXA, and the results showed that both forearm T-score and HU values could predict the cage subsidence following PLIF, but forearm T-score was a more accurate predictor.

In our study, logistic regression analysis revealed the forearm T-score and mean global HU value as independent risk factors for cage subsidence after PLIF (*P* = 0.016 and 0.031, respectively). Compared with the post-PLIF subsidence rate (30.2%) reported by Cho et al. [15], the subsidence rate (23.9%) in this study was relatively low, which may be related to our use of the pedicle screw system to improve posterior stability. The forearm T-score and mean global HU value of the 17 patients in the subsidence group were significantly lower than those of the 54 patients in the nonsubsidence group. Both forearm T-score and mean global HU value can predict fusion subsidence. Compared with the mean global HU value (AUC, 0.744; 95% CI, 0.544–0.943), the forearm T-score (AUC, 0.840; 95% CI, 0.672–1.000) was more predictive of cage subsidence. In our study, using a forearm T-score of − 2.6 as the threshold yielded a sensitivity of 84.6% and a specificity of 83.3%; with the mean global HU value threshold of 104.2 HU had a sensitivity of 84.6% and specificity of 66.4%. Therefore, patients with forearm T-score < − 2.6 were at a greater risk of cage subsidence after PLIF. Therefore, patients with forearm T-score > − 2.6 should be selected as candidates if spine surgeons avoid cage subsidence after PLIF.

Our study has some limitations. First, the single-center study design was retrospective in nature, the sample size was small, and follow-up time was short; thus, a large-sample long-term prospective study is warranted to validate our findings. Second, the study population included only patients with single-segment PLIF combined with bilateral pedicle screw fixation. Therefore, further investigation of patients undergoing other surgical modalities is required. Third, we did not discuss the correlation between cage size, cage position, intervertebral-space-correction height, and cage subsidence, many of which may lead to a mismatch between the vertebral endplate and cage, resulting in cage subsidence.

## Conclusion

Lower forearm T-scores and lower lumbar spine HU values were significantly associated with the occurrence of cage subsidence, with the lower forearm T-scores indicating a higher risk of cage subsidence than lumbar spine HU values. Forearm T-score is more accurate in predicting cage subsidence than spine T-score. Therefore, forearm DXA may be a fast, simple, and reliable method for predicting cage subsidence after PLIF. However, our results suggest that the degree of cage subsidence is not associated with clinical efficacy.

## Data Availability

All data analyzed during this study are included within the manuscript. The datasets used and/or analyzed during this study are available from the first author on reasonable request.

## Abbreviations

PLIF: Posterior lumbar interbody fusion
AUC: Areas under the ROC curve
BMD: Bone mineral density
BMI: Body Mass Index
DXA: Dual-energy x-ray absorptiometry
HU: Hounsfield Units
PACS: Picture archiving and communication system
ROC: Receiver operating characteristic curve
CT: Computed tomography
VAS: Visual Analog Scale
JOA: Japanese Orthopaedic Association
